# Providing sexual and reproductive health services to migrants in Southern Sweden: a qualitative exploration of healthcare providers’ experiences

**DOI:** 10.1186/s12913-022-08967-3

**Published:** 2022-12-21

**Authors:** Nada Amroussia

**Affiliations:** 1 Centre for Sexology and Sexuality Studies (CSS), Faculty of Health and Society (HS), Malmö University, Malmö, Sweden; 2 Department of Women’s and Children’s Health, Uppsala University, Uppsala, Sweden

**Keywords:** Sexual and reproductive health and rights, Migrants, Healthcare encounter, Qualitative thematic analysis, Person-centered care, Diversity

## Abstract

**Background:**

While a large body of research has focused on the challenges experienced by healthcare staff when providing sexual and reproductive health services, little attention has been paid to the ways healthcare providers navigate these challenges. This study examined healthcare providers’ accounts of encounters when providing sexual and reproductive health (SRH) services to migrants in Southern Sweden. It sought to examine challenges and dilemmas experienced by healthcare providers, strategies used to navigate these challenges and dilemmas, and assumptions underlying participants’ accounts.

**Methods:**

The data collection was conducted between September 2020 and March 2021. Qualitative thematic analysis was used to analyze thirty-one interviews with healthcare providers working in youth clinics and women healthcare clinics. The analysis was guided by a conceptual framework combining person-centered care approach, Foucault’s concepts on power/knowledge, and theories to navigate diversity in healthcare setting: cultural competency and cultural humility.

**Results:**

Three themes were identified in the analysis: 1) Between person centeredness and cultural considerations; 2) Knowledge positions and patient involvement; and 3) beyond the dyadic interaction healthcare provider-patient.

Some participants understood person-centered care as individualized care where the influence of culture on the encounter should be de-emphasized, whereas others tended to highlight this influence. Many participants viewed the influence of culture as primarily driven by migrants’ cultural backgrounds, and as a source of challenges and dilemmas. Participants’ strategies to navigate these perceived challenges and dilemmas included practicing cultural humility and seeking cultural competency.

Knowledge positions also emerged as an important aspect of participants’ accounts of encounters with migrants. Many participants experienced that migrant patients were lacking knowledge about the body and sexuality. This disadvantaged knowledge position affected migrant involvement in care.

Additionally, the study shows how participants placed their experiences in a broader organizational and social context. Participants highlighted several organizational challenges to encountering migrants and discussed dilemmas stemming from the interplay between migrants’ structural and individual disadvantages.

**Conclusions:**

The study findings illuminate the complex links between person-centered care and two important dimensions of the encounters with migrants: culture and knowledge positions. They also shed the light on the organizational and structural challenges surrounding these encounters. These findings suggest that multilevel strategies are needed to improve the quality of encounters when providing SRH services to migrants. These strategies could include ensuring universal access to SRH services to migrants, adjusting the encounter duration when interpretation is needed, and providing necessary resources to healthcare providers to build their structural competency.

**Supplementary Information:**

The online version contains supplementary material available at 10.1186/s12913-022-08967-3.

## Background

A large body of research has focused on the encounters between healthcare providers and migrants in the context of providing sexual and reproductive health^1^ (SRH) services.^2^ For instance, studies conducted in North America [1, 2], Europe [3, 4], and Australia [5, 6] pointed to different challenges experienced by healthcare providers. These challenges were mainly centered around the interactions between healthcare providers and migrants and included communication difficulties, perceived cultural differences, and lack of cultural competency, They were compounded by organizational difficulties such as time and resource constraints [1–6]. Similar challenges were also highlighted in studies conducted in Sweden [7–10].

Research on the encounters between healthcare providers and migrants tends to focus on the challenges, whereas little attention has been paid to the ways healthcare providers navigate or handle these challenges. Available research indicates that healthcare providers’ strategies to navigate challenges when providing SRH services to migrants include showing flexibility to patients’ preferred practices, building trustful relationships with migrants, as well as developing healthcare providers’ cultural competency and knowledge about other cultures [7, 11, 12]. Assumptions underlying these strategies are yet to be explored. Exploring these assumptions might provide a deeper understanding of healthcare providers’ perspectives and their ways of navigating the daily challenges encountered when providing SRH to migrants.

The aim of this study was to examine healthcare providers’ accounts of encounters with migrants^3^ when providing SRH services in Sweden. The study sought to examine: 1) challenges and dilemmas experienced by participants during the encounters, 2) their strategies for navigating these challenges and dilemmas, and 3) assumptions underlying participants’ accounts.

## Conceptual framework

In this conceptual framework, the encounter with migrants when providing SRH services is conceptualized as a space for implementing the Swedish ideal of person-centered care. It is also a space for negotiating power and navigating culture and diversity related issues. Healthcare encounter is understood as not only a temporally and spatially localized dyadic interaction between healthcare providers and patients, rather a more diffuse interaction that can often involve multiple actors and is localized in a broader organizational and social context [13, 14]. This conceptualization of the encounter helped examine the assumptions underlying participants’ accounts of challenges, dilemmas, and navigation strategies when providing SRH services to migrants.

### A person-centered care approach to the healthcare encounter

Despite the growing interest in adopting the person-centered care approach in Sweden and worldwide [15, 16], this concept (i.e., person-centered care) is still poorly defined [17] and therefore difficult to operationalize and implement. Nevertheless, different conceptualizations of person-centered care agree that this concept is grounded in a holistic view to care and marks a departure from the previous bio-medical and symptom-focused approach to care [15, 16]. An overview of reviews found that common components of person-centered care include shared power and responsibility between healthcare providers and the patients; trust and respect; empowering and getting to know the person, and communication [17]. Individualized care and personalized care have been often presented as important dimensions of person-centered care approach [16, 18, 19]. Andersson [16] stated that “person-centeredness builds on a fundamental respect of subjectivity, agency, capability and personhood”.

In this study, the focus was on the common features of person-centered care highlighted in previous literature that encompass, among others, holistic approach to care, person’s/patient’s agency, shared power and decision-making, and individualized care [16–19]. This conceptualization entails that the person/patient’s whole wellbeing is considered at the healthcare encounter including the person’s context, preferences, and beliefs. It also entails developing a partnership between healthcare providers and patients to “co-design and deliver personalized care” [18].

This conceptualization is particularly relevant to the Swedish context where person centered care is defined as an ethical standpoint guiding professionals’ practical actions and implying a partnership between patients, their relatives, and the professionals [20].

### Power dynamics in the healthcare encounter

The growing emphasis on shared decision-making in line with the person-centered care approach might be challenged by power relations in healthcare setting, and more specifically during the encounters. Knowledge plays an important role in framing these power relations. According to Foucault [21], power and knowledge are inextricably linked: “there is no power relation without the correlative of a field of knowledge, nor any knowledge that does not presuppose and constitute at the same time power relations”.

Foucault [22] described how medical knowledge turned into social privilege giving medical doctors the power to define reality and draw the boundaries between normality and deviance. In this context, medical knowledge operates as *a truth regime* where veridical truth can only be found in this type of knowledge [22]. This truth regime allows the distinction between true and false statements, determining how true and false can be sanctioned. It also determines the status given those who speak truth [23].

In the past twenty years, evidence-based medicine (EBM), increasingly privileged in healthcare settings, has emerged as the new truth regime [24]. Goldenberg [25] argue that while EBM was thought to challenge medical doctors’ authority, it has consolidated it through regulating the relation power/knowledge. For instance, EBM has been criticized for diminishing patient’s experiences and voices [26], and for creating a hierarchy of power/knowledge by deciding what knowledge is legitimate and what knowledge is not [24].

Drawing on Foucault’s concepts on power and knowledge, healthcare providers’ accounts were examined in the context of the recent shifts towards person-centered care and shared decision-making.

### Navigating diversity in the healthcare encounter

In the past few decades, *cultural competency* and *cultural humility* have emerged as prominent paradigms to address issues in healthcare setting stemming from the growing diversity of populations (e.g., language differences and perceived cultural differences). Both concepts paid a special attention to the influence of culture on the encounters between healthcare providers and culturally and ethnically diverse patients, such as migrants.

Cultural competency has different conceptualizations. One such conceptualization is developing the competence and knowledge to understand patients’ culture and cultural practices [27–29]. Cultural competency assumes that healthcare providers are able to “learn a quantifiable set of attitudes and communication skills” enabling them to work in a culturally diverse setting [30]. While this concept has been largely implemented in healthcare settings, it has been criticized for its failure to account for the fluidity of culture and for reinforcing stereotypes about minority groups [27–29].

Cultural humility was presented as an alternative to cultural competency that might allow “a deeper understanding of cultural differences in order to improve the way vulnerable groups are treated and researched” [29]. Tervalon and Murray-Garcia (1998) defined cultural humility as a process or “a lifelong commitment to self-evaluation and self-critique” and “to redressing the power imbalances in the patient-physician dynamic”. They argue that culture is not only restricted to dimensions like ethnic identity, but also encompasses other dimensions, such as the culture of the healthcare provider, that requires humility in working with patients and communities [28]. A concept analysis identified five attributes of cultural humility: openness, self-awareness, egoless, supportive interactions, and self-reflection and critique [31].

The concepts of cultural competency and cultural humility were applied to examine strategies used by healthcare providers to navigate perceived culture related issues when providing SRH services to migrants.

## Methods

### Study design

A qualitative methodology with an emergent design was employed in the study [32]. It sought to gain a deep understanding of the experiences and views of healthcare providers when providing SRH services to migrants.

### Study setting

The study site was the Scania County in Southern Sweden. Since 1960, the percentage of foreign-born residents in Sweden has been increasing steadily [33, 34]. In 2020, 19.7% of the Swedish population were foreign-born and 25.9% had a foreign background i.e., foreign-born residents or residents having two foreign-born parents [33]. In the Scania county, the total population is nearly 1,4 million inhabitants with foreign-born residents representing 22% of the population in 2019 [35]. The largest foreign-born groups are from the Nordic countries, the former Yugoslavia and the *Middle East*. In 2014, the implementation of person-centered care in this county was launched [36].

The Swedish Health and Medical Services Act guarantees health and healthcare services of good quality and on equal terms for the entire population [37]. The Swedish healthcare system is mainly publicly funded through taxation [38]. SRH services are provided by a network of centers and facilities organized at different levels including women healthcare centers and youth clinics. Women healthcare centers provide a broad range of healthcare services that differ by county. These services include childbirth services, prenatal care and postnatal care services, specialist gynecologist services, fertility treatment, and contraceptive counseling. Youth clinics provide SRH services and information to youth aged between 12 and 25 [39]. The services are free of charge for youth under the age of 18 and most of the clinics provide services free of charge for youth under the age of 25 [40]. In 2015, the Swedish Association of Local Authorities and Regions has launched the implementation of person-centered care approach in different counties. Person centeredness has become a core feature of the Swedish healthcare system [36].

In Sweden, entitlement to healthcare among migrants differs by migration status and requires being registered in the Swedish population register^4^ or being an EU/EEA citizen. This is except for people under 18 who are entitled to healthcare on the same terms as residents under 18 and registered in the Swedish population register. Asylum seekers and undocumented migrants are entitled to emergency healthcare and healthcare that “that cannot wait”,^5^ maternal healthcare, abortion care, contraceptive counseling, medication related to these types of care, and health examination (unless it is unnecessary). It is worth noting that the types of care to which asylum seekers and undocumented migrants are entitled differ by region and county [41]. In the Scania county, asylum seekers and undocumented migrants are entitled to the care listed above [42].

### Recruitment and data collection

The recruitment and data collection were performed in collaboration with a medical doctor pursuing a residency training in Obstetrics and Gynecology. The recruitment process targeted healthcare staff providing SRH services and working in public health centers in Southern Sweden, mainly women health clinics and youth health clinics. A combination of convenience, snowball, and purposive sampling strategies was used [43] to obtain a diverse sample in terms of demographic characteristics (gender, age, country of birth, and ethnicity), profession, and place of work. Using convenience sampling followed by purposive sampling, potential participants were contacted directly the medical doctor through face-to-face contact and via electronic mail. Flyers were distributed in some healthcare centers and through emails. Using snowball sampling, some potential participants were contacted after being referred by interviewed healthcare providers. In addition to flyers, an information sheet was used to invite potential participants. It contained data collection tools used i.e., recorded interviews and pre-interview questionnaires, preliminary topics to be explored, and measures to ensure participants’ confidentiality.

The data collection was performed between September 2020 and March 2021 and was adjusted to the Covid pandemic. In-depth interviews were conducted at the participants’ places of work and through the applications Zoom or Microsoft teams when using face-to-face interviews was not possible. The interviews were guided by a semi-structure interview guide with open ended questions and addressed four main topics: 1) participants’ views on working in a culturally diverse setting, 2) challenges and dilemmas when providing SRH services to migrants, 3) daily strategies to navigate challenges and dilemmas, and 4) suggestions to improve healthcare providers’ work experiences (appendix 1). The interview guide was adjusted after conducting three pilot interviews included in the analysis. The adjustment resulted into minor changes, including reformulating some questions to ensure clarity and adding follow-up questions.

Interviews were conducted in Swedish and English by the first author and the medical doctor. One interview was conducted in Arabic. Interviews lasted on average 50 min and were audio recorded. The data collection continued until saturation was reached i.e. estimating that no new information or themes emerged during the interviews [44]. In this study, saturation was considered as reached when similar accounts were shared by participants, and when no new information related to challenges and dilemmas, and strategies to navigate them emerged in the last interviews as compared to the previous interviews.

### Sample

A total of thirty-one participants were interviewed in the study (Table 1). The majority were women (27 out of 31), Swedish- born (20 out of 31), and self- identified as Swedish (20 out of 31). Participants’ ages ranged from 25 years old to 63 years old with twelve participants were aged between 25 and 35 years old, and fourteen participants were aged over 45 years old. Participants’ professions were medical doctors (10), midwives (7), nurses (8), assistant nurses (2), counselors (3), and one participant had a managerial position. More than half of participants (16 out of 31) reported receiving special training in sexology and sexual health, and only three participants reported receiving special training in working with migrants.

**Table 1:** Participants’ characteristics

Characteristics	Number of participants
**Gender**	
Man	04
Woman	27
Other	00
Age^*^ (years)	
25–35	12
36–45	04
46 or older	14
Self-identified ethnicity^*^	
Swedish	20
Non-Swedish	05
Both/Mixed	05
Place of birth^*^	
Sweden	20
Abroad	10
**Profession**	
Medical doctor	10
Midwife	07
Nurse	08
Assistant nurse	02
Counselor	03
Manager	01
Special training in sexology^*^	
Yes	16
No	14
Special training in working with migrants^*^	
Yes	03
No	27

^*^ The total number is 30 instead of 31 because there is one missing information

### Data analysis

Interviews were transcribed verbatim by the author N.A., the medical doctor pursuing a residency training in Obstetrics and Gynecology, and a professional transcriber. After transcribing the interviews, the analysis process was undertaken by the author N.A who used a qualitative thematic analysis following Braun and Clarke approach [45].

The first analytic step consisted in reading the interviews thoroughly with taking notes and writing preliminary interpretations. The second analytic step consisted in coding and developing themes. A combination of inductive and deductive approaches was used. The first interviews were coded inductively. The software NVivo (released in March 2020) was used for coding. The initial codes were used to code the rest of the interviews with adding new codes generated during this process [45]. Codes were examined to look for thematic patterns in the data. Preliminary semantic themes were grouped in two groups: 1) Challenges and dilemmas; and 2) navigation strategies. Using the conceptual framework, latent themes related to the assumptions underlying these challenges, dilemmas and navigation strategies were, then, identified and grouped into a third group “assumptions”.

The third analytical step consisted in examining the three groups. Preliminary semantic themes related to 1) challenges and dilemmas, and 2) navigation strategies were rearranged within the assumptions’ group, which allowed focusing on the emerging latent themes. The last analytical step consisted in reviewing, contrasting, and refining the latent themes to obtain three final themes (Appendix 2). The final themes were discussed with senior researchers and presented in two seminars. The analysis was conducted in English; and quotes presented in this article were translated to English when needed.

### Ethical considerations

The Swedish Ethical Review Authority approved the study (Dnr: 2020–01,043). Prior to interviews, a consent script including information about the study aim and procedure was sent to all participants. A verbal informed consent for interviews and audio-recordings was obtained from all participants. Pseudonyms were used to ensure participants’ confidentiality and to de-identify interview data. Only the research team had access to the recordings that were stored in a USB flash drive disconnected from the web. The participants’ identifying information (name, place of residence…) were removed from the transcripts. No identifying information were collected on the pre-interview questionnaires (e.g., participants’ names…).

## Results

Three themes were identified and presented in this paper: 1) Between person centeredness and cultural considerations; 2) Knowledge positions and patient involvement; and 3) beyond the dyadic interaction healthcare provider-patient.

### Theme 1: Between person centeredness and cultural considerations

From across many interviews, it appears that adopting a person-centered care approach and considering the influence of culture on the encounters are not always compatible. These two seemingly incompatible approaches resulted into different ways of framing dilemmas and strategies to navigate the healthcare encounters with migrants.

### Subtheme 1: Person centeredness

The person-centered care approach was prioritized by many participants who often understood person centeredness as providing individualized care where the focus is on meeting the need of the individual. These participants tended to de-emphasize the role of culture in the encounter when providing SRH services and related the discussion on the influence culture to categorizing patients, generalizing, considering group membership, or even prejudices. Therese (Swedish-born, medical doctor) said:

“It is better to just go and meet the person than projecting some form of prejudice from the beginning. I don’t know… it is fun to learn about other cultures and other values. But I think in my professional role it can probably be better that you just go in without prejudice at all, and just see the individual”.

Echoing Therese, Inga (Swedish-born, assistant nurse) related de-emphasizing cultural differences to treating patients equally regardless of their background:

“So, I don’t see different cultures… I don’t see if a woman is Swedish or foreign-born, I don’t recognize it… I don’t have it… everyone is equal to me”.

Some participants endorsed the individual variabilities in their interactions with migrants and refrained from providing a generalized picture of the encounters. Participants also perceived that the preferred way to encounter migrants was to focus on the patient as an individual beyond group membership. They found that this way can mitigate the influence of their prejudices on the interaction. Kristina (foreign-born, midwife) described her experience of providing contraceptive counseling to migrants by saying:

“I think you have to start by asking questions because if I start right away and treat different ones based on my pre-assumptions or prejudices, then, I think we end up wrong, right away. And it’s again, who am I meeting as an individual”.

In this way some participants described their encounters with migrants and expressed their concerns about having prejudicial attitudes.

### Subtheme 2: Navigating culture-related dilemmas and challenges

In contrast with the tendency to minimize the influence of culture on the encounter, many participants highlighted its role. In doing so, participants often emphasized the influence of patients’ cultural backgrounds and non-Swedish cultures. Julia (Swedish-born, medical doctor) described the patient’s cultural background as an extra dimension to be considered in communicating with patients:

“So, you need to find the right level (…) with all the patients you meet. It becomes like an extra dimension when there is another culture involved. (…) If it’s also a different culture, a different background, or a different country, a different system, then, it becomes like an additional dimension, I would say.”

The influence of culture was not always described in a neutral way by participants. Some participants viewed this influence as one of the barriers in providing SRH services to migrants. Participants also emphasized that several dilemmas encountered when working with migrants are rooted in the patient’s culture. An example of these dilemmas is patient preference for healthcare providers of same gender. In this context, it refers to migrant women preferring being examined by female healthcare providers when seeking, among others, maternal healthcare services. Many participants considered this issue as a barrier to care and a source of conflict in the encounter. They often attributed it to cultural explanations, or “a different culture” as stated, for example, by Ida (foreign-born, assistant nurse):

“It’s common for foreign-born patients when they seek healthcare, when they find out it's a male assistant nurse, they become a bit shy; and you can't take that away from them, and especially the newcomers, because they have a different culture”.

Another dilemma that was often framed as cultural was migrant youth’s views on sexuality, including views on virginity, sexual desire, and masturbation. These views were considered as shaped by youth cultural background. Bodil (Swedish-born, counselor) described the difficulty of addressing migrant youth views on masturbation by saying:

“Considering masturbation as harmful, for example, self-sex. We have talked and talked about it here. But it is difficult…because (…) it’s so ingrained and doesn’t depend on just school knowledge rather it’s considered by many as culturally and religiously extremely important facts”.

Some participants brought up what they called honor issue as a challenge when encountering migrant youth or migrant women seeking, for example, abortion services. They described how honor issue could manifest into different forms of pressure from the family on aspects of youth sexual life (e.g., being sexually active, accessing or using SRH services, disclosing sexual identity) and on women’s decision to have an abortion. Although some participants mentioned that this issue was not specific to migrants, participants often noted that it is more common when encountering migrants. Lena (foreign-born, midwife) described one of the challenges encountered when working in a youth clinic by saying:

“Previously, the youth clinic was located in a much more immigrant-dense area; and we often encountered the honor problem. You can’t say at home that you have boyfriend, girlfriend or partner (…) Yes, the honor problem was much more often encountered.”

Participants related this issue to what they defined as honor culture that could be more encountered with migrants. Johanna (Swedish-born, midwife) said:

“It’s not uncommon to encounter elements of honor culture… with… foreign-born. Absolutely, as many come from the part of the world where there are strong traditions around the family and family values ​​and what is a family and what is allowed in a family or in a marriage, or what is not allowed. It's pretty common anyway.”

Sometimes, participants discussed the influence of culture within the dichotomy western culture vs. non-western culture where western culture was often perceived as less visible and less problematic. Jacob (foreign-born, medical doctor) said:

“If one talks about foreign-born patients, then, it depends on where they come from. I have never experienced a problem if I meet women from European countries (…). But it’s most problematic when you have… patients from Afghanistan and other Arab countries… who are Muslim; and then, if they come with… their husband, they do not want male doctors to examine them.”

Participants reflected on different strategies to address what they perceive as culture related challenges and dilemmas. These strategies can be summarized into *practicing cultural humility* and *seeking cultural competency*. Although participants might not be aware of the concept cultural humility, this concept was reflected in their described attitudes and practices when providing SRH services to migrants. They highlighted the importance of being open and humble when interacting with migrants. According to participants, humility and openness entailed asking open questions to patients and avoid pre-assumptions. Sophie (Swedish-born, midwife) shared her view on how to provide SRHR-related information to migrant youth by saying:

“By trying to ask them what they know because then you can explain… because, as I said, it’s easy to just appear and just talk and inform without knowing what they already know or what they have previously as misconceptions. (…) In that way try to give information, but also, you have to be… try to be quite careful and humble. You have learned in one way, and I have learned in another. So that you do not end up in like “I know best, and you know nothing””.

Self-reflection and self-awareness were also mentioned by some participants as ways to navigate working in a culturally diverse setting. Karin (Swedish-born, counselor) described the process of self-reflection by saying:

“You usually have, before you meet a person, most of us have a pre-assumption (…). But then, you know that you have it. But you can also ask questions both to yourself, i.e., silent questions, but also to the patient…”

Participants also expressed their need to develop their competencies in meeting migrants and patients having different cultural backgrounds. They focused particularly on their need for increasing their knowledge about different views on sexuality in different countries and cultures.

In addition to these strategies, some participants brought up ethnic matching and demographic representation as a way to overcome what they perceived as culture related dilemmas. Ethnic matching and demographic representation refer to recruiting healthcare providers having similar backgrounds with patients. Foreign-born participants often described how they had better experiences when encountering migrants in comparison with Swedish-born healthcare providers. Ella (foreign-born, medical doctor) said:

“It's also something I've heard several…many times…from patients, that it’s nice for them to see a doctor who is not Swedish (…) because they know that I had also difficulty in the beginning with the language and so on. So, you can feel a little alike.”

This positive experience was shared by some other foreign-born participants who considered having a foreign background as a facilitator when working with migrants.

### Theme 2. Knowledge positions and patient involvement

Many participants described how migrants had limited or lacked sexuality-related knowledge and what they considered as “basic knowledge” about the body. Lukas (Swedish-born, nurse) said:

“If you think about the basic ... the basic sexology or sexual education ... that ... those who were born in Sweden receive, is on a completely different level than the one of migrants. What I see as basic knowledge is not at all certain to be known by migrants”.

While some participants mentioned that the lack of knowledge was not specific to migrants, it was often brought up as one of the main barriers in communicating with migrants and a source of frustration in the encounter. To describe her last encounter with a migrant woman, Helena (Swedish-born, medical doctor) said:

“She was 53 and… she did not have much knowledge about reproductive health; but she had a big cyst, and it was quite a challenge to try to explain to her because she had absolutely no idea how her body worked.”

Many participants emphasized relying on facts and evidence-based information when interacting with migrants. A contrast between healthcare providers’ evidence-based discourse and migrants’ nonfactual discourse appeared in some interviews. For instance, some participants used the term “myths” to refer to migrants’ views on sexuality-related matters or to their conceptions of the body. Agnes (Swedish-born, medical doctor) shared:

“It's also difficult when you don't know what they [migrant women] do know, and you don't know what their idea behind how the body works is like. If I would know her [a migrant woman] myth, her concept of how the body works, I could work with that and explain in her terms…in terms she understands what is going on here.”

One mentioned “solution” to the perceived lack of knowledge about sexuality and misconceptions about the body is what can be understood as *educating the migrant* which consists in educating the migrants in matters related to SRHR as well as to the Swedish culture and system. Some participants also mentioned using visual communication (pictures, drawing) to facilitate communication with migrants and to overcome difficulties stemming from the lack of sexuality-related knowledge. Helena (Swedish-born, medical doctor) said:

“I always have a small sketch that shows what the uterus and ovaries and fallopian tubes look like. I often keep it in front of me and show what it looks like. This is the vagina, and this is the ovaries that make eggs.”

Migrants’ disadvantaged knowledge position was reflected in the power dynamics in the healthcare encounter. Some participants mentioned how this lack of knowledge led to migrants’ passiveness in the encounter contrarily to the well-informed Swedish-born patients who were perceived as more proactive and more involved in decision-making. Mariam (foreign-born, medical doctor) described the difference in encountering foreign-born patients and Swedish-born patients by saying:

“I think they are much more educated, Swedish patients than non-Swedish. They are more informed, and they dare to question things. They would like to have a plan on how to proceed in different situations compared to foreign-born. They [foreign-born patients] …do not ask anything…they accept everything. They…do not dare to tell anything.”

Some participants recounted their attempts to encourage migrant participation in care. Ayman (foreign-born, medical doctor) reported how he attempted to encourage migrants to be involved in decision-making:

“I always try to say that here it’s better that you… participate in decisions. It's your body. (…) It improves the treatment results in that way. But I don’t know if I managed to convince them or not.”

Many participants described how navigating different dilemmas and barriers requires involving and discussing with the patient as a participant in decision-making. In describing her way of navigating the issue of what she described as honor culture when providing abortion counseling services to migrant women, Johanna (Swedish-born, counselor) said:

“I talk to her [a migrant woman] about how… there is a support and help to get if she has a desire or wants to get out of this. I can also talk about… her thoughts and feelings (…) and my goal is not to persuade anyone, rather that I should show existing alternatives, if she wants to change something or… I try to find out her thoughts and wishes.”

As illustrated by these examples, from across interviews, it seems that attempts to encourage migrants’ involvement in care, in line with the person-centered care approach, can be challenged by migrants’ disadvantaged knowledge position.

### Theme 3: Beyond the dyadic interaction healthcare provider-patient

Participants viewed their encounters with migrants as not only a dyadic interaction healthcare provider-patient. They often placed their encounters’ experiences in a broader organizational and social context moving beyond the traditional conceptualizations of healthcare encounters.

### Subtheme 1: Organizational challenges to encountering migrants

Language difficulties were the most commonly barrier in communicating with migrants described by participants. Language difficulties were often mentioned as an explanation for unequal care to migrants as they can restrict migrants’ ability to express their needs and “stand up for themselves” (Linnea (Swedish-born, nurse)). These language difficulties seem to be exacerbated by the time constraint in the encounter where the duration is the same regardless of patient’s background. Many participants described how interactions with a patient who did not speak Swedish, including interactions through interpretation, were time consuming. This could be particularly relevant for patients with lower educational level. Eva (Swedish-born, midwife) said:

“I used interpreter quite a lot … And we have the same time for appointments… and during like…40 minutes or one hour…it’s the same for all; and of course, it takes much longer time to have an interpreter, and if this woman is not…if she’s illiterate…I mean, of course, it takes longer time to explain everything.”

To address language barriers, the Swedish health system offers interpretation services that were viewed as a challenging solution. Participants often complained about the quality of the interpretation services especially telephone interpretation, the unavailability of these services for some languages, and the lack of professional medical interpreters able to translate medical terminology. Some participants also described the difficulty of relying on an interpreter when discussing sensitive topics with patients. Participants recounted how discussing sexuality-related matters could be uncomfortable for both patients and interpreters.

Although interpretation services were often available for the most commonly used languages, participants noted that these services were not always accessible in case of emergency, which forced them to rely on their bilingual colleagues or on patient’s relatives or family members. Participants perceived using relatives or family members for interpretation as particularly problematic. They described how it could lead to breaching patient confidentiality. Participants also reported how relatives could interfere in patients’ decisions. The concern of interfering with patients’ decisions or influencing their views was also present when relying on interpretation services as expressed by Bodil (Swedish-born, counselor):

“We have such a terrible example. It didn’t happen to me. But we had an interpreter who told a young person that his sexual orientation was a sin, that he would be punished with death and all that. Luckily, our staff picked up that it was something strange and found out what had been said. But it creates distrust in the conversations”.

Many participants viewed that an organizational change is needed to overcome communication issues related to language difficulties and interpretation. Participants mainly stressed the importance of allocating more time which can improve the encounters’ quality and ensure meeting migrants’ needs. Anna (foreign-born, midwife), for example, emphasized the importance of allocating more resources and time to improve the encounter with migrant youth by saying:

“But we need more time and more resources. I think we can do everything if only we get more resources ...to better network with many different actors [the participant named local organizations who provide services to migrants].”

As shown above, addressing organizational challenges seems to fall beyond healthcare providers’ role and capacity. Few ways to navigate these challenges were described by participants who mainly focused on presenting suggestions to overcome them.

### Subtheme 2: Handling the interplay between migrants’ structural and individual disadvantages

Participants described how their encounters with migrants were shaped by migrants’ multiple disadvantages at the structural and individual level. These disadvantages were viewed as interrelated and as sources of different challenges and dilemmas in the encounters ranging from communication challenges to difficulties to ensuring equal care.

At the structural level, participants viewed migration processes in the host country as not only a stress factor that might negatively influence the encounters, but also as a source of vulnerability restricting migrants’ right to health and their access to SRH services. Alice (Swedish-born, nurse) said:

“There are probably no as many people who fall between the chairs as those who are during a migration process (…) I know that it’s clear who should take responsibility, but it’s not so clear in practice (…) what you are entitled to when you are during the process, and what type of care you are not entitled to.”

Alice continued explaining how financial costs of care are defined by the migration status , which interferes with the financial accessibility of some SRH services and can represent a dilemma for healthcare providers willing to provide care for those who are in need:

“It's really mostly about who is liable for payment and how to pay, depending on what type of migration process you are in, if it’s about seeking asylum or if it's about immigrating here. Then, you usually have a few different opportunities… to seek care. (…) Then, for those who cannot pay, but you want them to receive care, you don’t know how to get around it properly; and how to give them the care they need.”

Participants mentioned how the issues of financial accessibility and restricted access to SRH services were particularly relevant for some groups of migrants, namely undocumented migrants. Concern about the accessibility of SRH services was also voiced by healthcare providers working in youth clinics. However, in this case, the issue of accessibility was not driven by financial costs rather by the centralization of the youth clinic services and the difficulty to reach out to migrant youth. Sophie (Swedish-born, midwife) said:

“We also talked about…we have a big clinic in the center. We have talked for many years about trying to have some kind of branches out, more like in the outer edges;(...) I think it can be good, because if we get to show ourselves out there and be in their environment a little bit, it would also be easier for them to come here to the center. We believe that…or we know that many who live in the suburbs never come to the center.”

Another facet of migrants’ disadvantages brought up by participants is the exposure to forms of what can be understood as *overt or covert racism* in healthcare setting. For instance, some participants pointed to the prejudicial attitudes of their colleagues; and a very few of them recalled witnessing incidents in the past where their colleagues directed what they viewed as explicit racist comments towards migrants. Some participants viewed the issues of prejudices and hostility as barriers in the healthcare encounters with migrants. Katarina (Swedish-born, nurse) summarized what she perceived as barriers in these encounters by saying: “I said language, and I said prejudices and discrimination.”

Some participants also related these issues to receiving unequal care to migrants. Inga (Swedish-born, assistant nurse) said:

“I want to believe that everyone receives equal care. It's my wish and it's what I want and hope and believe. But I suspect that isn’t really the case. I think that the hostility and prejudices have negative effects. I think that they [foreign-born patients] may go home earlier, and that they don’t really receive the same care that a Swede… had received.”

This view was shared by Eva (Swedish-born, midwife) who said:

“NA: And based on your experience, do you think that migrants are receiving equal care?

Participant: No! Not as much I would like to…

NA: Why?

Participant: () I also think many colleagues…I mean we have (very short silence) prejudices. I am really sad to tell it because it’s really like that…”.

While most of these participants attributed the issue of prejudice to individual attitudes, Marie (Swedish-born, manager) pointed to the structural nature of this issue. She also noted that this issue can represent a concern for healthcare providers working in youth clinics who might feel compelled to make extra efforts to address it and ensure equal care for migrant youth:

“We want it [that young migrants are receiving equal care]. But I know…on the other hand, maybe not always and that’s nothing that I am proud of (…) maybe we don’t try that hard sometimes, but it’s nothing that I meet. (…) I mean…when for example, you look at people that are looking for jobs…and you have your name that is Mohammed, Mustafa, a foreign name… it’s really hard to come to an interview. But (…) if you have a name like me, it’s much easier, if you compare. So, of course, I am in this society too. (…) But we try a lot to not be like that.”

These structural factors intersect with migrants’ individual-level disadvantages including education and age. Some groups such as older migrant women and migrants with lower education were perceived by participants as particularly vulnerable. Older migrant women were mostly described as having more language difficulties, more difficulties to navigate the Swedish healthcare system, and being more influenced by their home-country culture as compared with other migrants. Participants felt that older migrant women’s disadvantages undermined the quality of the encounter. Some participants described the difficulty of providing SRH services to older migrant women and contrasted these experiences with their experiences when providing SRH services to younger migrants. They felt that it was easier to interact with migrant youth who were viewed as more integrated in terms of language and understanding of the Swedish culture, which was reflected in their views and attitudes towards SRHR-related matters. Kerstin (Swedish-born, midwife) pointed to the age differences in attitudes towards abortion among migrant women by saying:

“I think we have a lot of foreign-born young people where the culture doesn’t allow, for example, an abortion, but they still come and do an abortion. They are very open about it. While we have many older women where I notice a completely different… shame about it, (…) because, as I said, many younger are more open to it, although perhaps the culture doesn’t allow it.”

Educational level stands out as the one of the most important factors shaping the encounter with migrants when providing SRH services. Participants related educational level not only to the ability to learn Swedish and navigate the Swedish healthcare system, but also to the access to SRHR-related information, the level of knowledge about the body, and to views on sexuality-related issues. Lower educational level was perceived as a disadvantage and a barrier in the encounter. For example, Ella (foreign-born, medical doctor) said:

“Yes, I think that lack of education, for example, sometimes not only they [migrants with lower education] can’t speak Swedish, but sometimes they can’t communicate well because they have never… maybe never studied anything. So, they don’t really understand how the body works and what does man when you say "uterus", "placenta", you know? So, it's also a lot that they don’t have a good idea of ​​how their period is and so on”.

This example shows how the intersection of being migrant and having lower education negatively impacts the interaction healthcare providers-migrants. Lower education cannot only be considered as an individual disadvantage as it can reflects structural disadvantages e.g., access to education in the home country.

## Discussion

The study findings illuminate the complex relations between person-centered care, culture, and knowledge positions that underlay participants’ accounts of challenges, dilemmas, and navigation strategies when providing SRH services to migrants. They also point to the structural and organizational challenges affecting the encounters between healthcare providers and migrants.

Some participants viewed person-centered care as individualized care with little room for considering the influence of culture, whereas others emphasized the role of culture when encountering migrants. The first view on the role of culture seems to be primarily driven by the fear of reinforcing stereotypes about migrants in Sweden and might be understood in light of the discussion on cultural otherness in the Swedish context. Cultural otherness refers to framing migrant issues solely within the culturalist framework without considering other salient socio-economic factors, along with reinforcing cultural hierarchy [46]. In this sense, participants seem to be willing to distance themselves from the cultural otherness discourse by minimizing the role of culture in their encounters with migrants. Nevertheless, this view might not be in line with conceptualizations of person-centered care that emphasize seeing the person as a whole and considering the person’s preferences and beliefs [18]. While previous literature focused mainly on the concept of patient centeredness and not person centeredness, it suggests that patient centeredness and diversity related concepts such as cultural competency have synergetic effects and can help improve the quality of healthcare services provided to culturally and ethnically diverse patients [47–49].

Participants who discussed the influence of culture on their healthcare encounter experiences tended to reflect primarily on migrants’ cultural background. This approach to discussing cultural diversity in healthcare setting is well documented and criticized as it assumes that the problem is the patient’s culture that needs to be addressed to ensure effective healthcare [50, 51]. To navigate the perceived culture related dilemmas, participants adopted different strategies. Some of these strategies, such as developing cultural competency and increasing healthcare providers’ knowledge about other cultures, were reported in previous studies [7, 12]. The current study adds to this literature by showing how healthcare providers incorporated some core components of cultural humility, including openness and self-reflection, in their daily encounters with migrants.

Other important insights in this study relate to the role of knowledge in shaping healthcare providers’ interactions with migrants when providing SRH services. In their encounters, many participants experienced that migrant patients were lacking knowledge about the body and sexuality. This finding might have different explanations. By referring to Foucault concepts on power/knowledge and regime of truth [22], this finding might raise a question about what type of knowledge was considered as legitimate and valid in these healthcare encounters, given the normative authority of medical knowledge and EBM as regimes of truth. This finding might be also understood by considering the assumptions underlying the new patient’s role. As suggested by Sulik and Eich-Krom [52], terms such as personal responsibility, rationality, proactive and prevention-conscious behavior are used to define the new role of patients. These expectations might have contributed to framing participants’ views regarding migrants’ knowledge positions.

Some participants even linked this perceived lack of knowledge to migrants’ passiveness and lack of involvement in care and decision-making. This lack of involvement can be explained by migrants seemingly disadvantaged knowledge position. Confidence in the value of own knowledge and ability to acquire medical knowledge were highlighted as facilitators to shared decision-making in healthcare [53]. Lack of migrants’ involvement can also be explained by healthcare providers’ views. Healthcare providers’ views that patients lack medical knowledge can be a barrier to their involvement in decision-making [54]. It might also reflect migrants’ unfamiliarity with the Swedish healthcare system where patient involvement in care is central.

Power dynamics in the encounters cannot be separated from the social context surrounding these encounters. Groups with historically less power, such as women, minority groups, might lack resources to challenge medical authority [22, 55]. This can be also relevant for migrants who are experiencing multiple disadvantages that might hinder their involvement in healthcare.

Finally, the study findings shed the light on challenges and dilemmas faced by healthcare providers beyond the dyadic interaction healthcare provider-patient. Interestingly, participants framed these organizational and structural challenges as not only a difficulty in their work, but also as a source of unequal care for migrants. This shows that the participants in this study were sensitive to the complexity of factors shaping migrants’ healthcare experiences. In addition to the role of factors related to communication issues (e.g., language barriers, the encounter duration…) highlighted in previous studies [3, 4, 8], healthcare providers drew attention to the determinantal effects of prejudices on the quality of encounters when providing SRH services to migrants. Such effects were also outlined in previous literature [56]. Nevertheless, the issue of racism in Swedish healthcare is still under-studied and difficult to research [57]. Generating evidence on the experiences and views of prejudices and racism from healthcare providers’ and patients’ perspectives are needed to improve the quality of care for diverse populations.

Healthcare providers also pointed to the role of structural factors, mainly migration policies in restricting some groups of migrants’ access to SRH services. By referring to Metzl’s and Hansen’s [58] approach to structural competency, the awareness of structural factors affecting migrant’s health and access to SRH services might be a first step towards developing healthcare providers’ structural competency. Neff et al. [59] argue for the importance of incorporating structural competency in the training of healthcare providers to prepare them for understanding and addressing structural challenges affecting patients’ health.

### Methodological discussion

The findings of this study derived from interviews conducted in Southern Sweden where the proportion of foreign-born residents and residents with foreign background is relatively higher than the average proportion in Sweden [33, 35]. Participants in this study encountered migrants on a daily basis in their work and might be ahead in developing strategies to navigate challenges and dilemmas that might arise during the encounters. Although the study findings might not capture the experiences of healthcare providers in all Sweden or in other high-income countries, the study findings might be tranferable to similar setting and might inform the provision of SRH services to migrants in these settings.

Additionally, more than half of the interviews used in this study were conducted via the applications Zoom or Microsoft teams as a consquence of the Covid pandemic. Synchronous online interviews were used as they were close to face-to-face experiences [60]. While online interviewing facilitated the data collection process and offered more flexibility to participants who were able to join the interviews from home or workplace, it raised the issue of ensuring participants’ confidentiality. Univited persons might access the virtual room [61]. To reduce this risk, participants were offered a link protected by a password where only the researcher can allow the users to join the interview. Finally, quotes were translated from Swedish when needed. The process of translation might have led to losing the meaning of some contextual expressions. 

## Conclusions

This study contributes to the understanding of healthcare providers’ experiences of encountering migrants when providing SRH services. Participants’ accounts of challenges, dilemmas, and navigation strategies are shaped by their assumptions regarding person-centered care and the role of culture in the encounter as well as their views on migrants’ knowledge. A deeper exploration of the relation between person-centeredness and culture within SRH services can ensure that healthcare providers are better equipped to meet the needs of patients with different backgrounds. The study also draws attention to the role of knowledge positions in shaping power dynamics in the encounter by constraining migrants’ involvement in care.

Additionally, healthcare providers’ encounters with migrants were influenced by organizational and structural challenges beyond the dyadic interaction healthcare provider-patient. This suggests that multi-level strategies are needed to improve healthcare providers’ and migrants’ encounter experiences, the quality of SRH services, and ultimately migrant SRH. Examples of these strategies could include ensuring universal access to SRH services to migrants regardless of their migration status, adjusting the encounter duration when interpretation is needed, and providing necessary resources to healthcare providers to build their structural competency.

## Supplementary Information

**Additional file 1:** **Appendix 1. **Interview guide.** Appendix 2. **Analysis process.

## Data Availability

The dataset analyzed during the current study is not publicly available to ensure participants’ confidentiality but is available from the corresponding author on reasonable request.

## Footnotes

1 The Swedish national strategy for sexual and reproductive health and rights adopted the integrated definition of sexual and reproductive health and rights provided stated in the Guttmacher-Lancet commission report 1. Starrs AM, Ezeh AC, Barker G, Basu A, Bertrand JT, Blum R, et al. Accelerate progress—sexual and reproductive health and rights for all: report of the Guttmacher–Lancet Commission. The Lancet. 2018;391(10140):2642-92. According to this report, sexual and reproductive health is defined as “a state of physical, emotional, mental, and social wellbeing in relation to all aspects of sexuality and reproduction, not merely the absence of disease, dysfunction, or infirmity.”

2 According to Guttmacher-Lancet commission report 1. Ibid., SRH services refer to “accurate information and counselling on sexual and reproductive health, including evidence-based, comprehensive sexuality education; information, counselling, and care related to sexual function and satisfaction; prevention, detection, and management of sexual and gender-based violence and coercion; a choice of safe and effective contraceptive methods; safe and effective antenatal, childbirth, and postnatal care; safe and effective abortion services and care; prevention, management, and treatment of infertility; prevention, detection, and treatment of sexually transmitted infections, including HIV, and of reproductive tract infections; and prevention, detection, and treatment of reproductive cancers.”

3 In this study, the term *migrants* is used to refer to foreign-born residents who migrated to Sweden.

4 It requires a valid residence permit.

5 Healthcare that cannot wait is interpreted case by case by the medical doctor or the dentist.
