# Supplementary material for: Providing sexual and reproductive health services to migrants in Southern Sweden: a qualitative exploration of healthcare providers’ experiences
Source: BMC Health Serv Res. 2022 Dec 21;22:1562. doi: 10.1186/s12913-022-08967-3 (PMC9768979; doi:10.1186/s12913-022-08967-3)
Supplement: Supplementary file 1 — Additional file 1: Appendix 1. Interview guide. Appendix 2. Analysis process. [file 12913_2022_8967_MOESM1_ESM.docx]

**Appendix 1: Interview guide**

**Introduction**

Thank you for volunteering to take part in this study. I appreciate your time and effort to be with me (us). My name is .................................... I have some questions about your experiences with providing sexual and reproductive health services in Sweden. I would like to understand the barriers and the facilitators to providing SRH services in a multicultural setting. The sexual and reproductive health care services enclose family planning services, information and counselling; prenatal care, delivery and post-natal care services; prevention and treatment of IST including HIV; treatment of reproductive system cancers and breast cancer; and abortion services; and prevention and treatment of infertility. I am also interested in knowing more about the complexity of engaging migrants and especially migrant youth in these services.

Before starting the interview, I would like to remind you that there are no right or wrong answers to any of the questions that I ask. All your experiences and opinions are valid. Your participation is completely voluntary. You can stop your participation at any time and you have the right to not answer or skip questions if you want to. We will audio-record our discussions and the recording will be transcribed. However, only the research team will have access to the recording. We encourage you to use pseudonyms to protect your confidentiality. We will not associate any identifying information (name, place of work…) with the recording or transcripts.

**Would you agree to record the conversation?**

1. Could you present yourself ? What services you are involved in?

**Participants’ understandings of the challenges and facilitators of providing sexual and reproductive health services to migrants**

1. Think about your last encounter with a migrant. Could you tell us more about this experience ?
2. How do you describe this experience ? Why ?

- Probe : have you had any difficulties ? Why ?
- What do you think has worked well ? How ?

1. In which way your last experience was similar or different from your other encounters with migrants ?

- What about your experiences with migrant youth ?

1. Have you faced any barriers when working with migrants ? Could you tell us more about that ?

- Have you faced similar or different barriers when working with migrant youth? Could you tell us more about that ?
- How have you tried to handle these barriers? Did that work in your opinion?

1. Working with migrants can be seen as working in a multicultural setting. Is it something that you have experienced?

- How do you view a multicultural setting?
- Have you faced any dilemmas when working in a multicultural setting in the individual encounters ? Could you tell us more about that ?
- Have you faced similar or different dilemmas when working with migrant youth? Could you tell us more about that?
- How have you tried to handle these dilemmas? Did that work in your opinion?

1. I am interested in knowing about the similarity in working with migrants and Swedish-born people. But I am curious to know what you consider as a migrant.

- Could you tell me about the similarity in working with migrants and Swedish-born people ?
- What about differences ?
- What about migrant youth ? Are these differences relevant when you compare your working experiences with migrant youth and Swedish-born youth ?

1. Is there any similarity or difference in working with different groups of migrants?

- What about migrant youth ?

1. How do you think the patient’s background influences the encounter?

- for example :
- Country of origin
- Education,
- Religion,
- Migration status (undocumented vs. documented or refugee..),
- Duration of stay in Sweden.
- Could you elaborate more on that ?
- Would you say that this is relevant to migrants in general or it is specific to migrant youth ? How ?

1. Do you feel that some factors or conditions can facilitate your work with migrants ?

- Could you tell us about that ?
- What about migrant youth ?

1. Do you feel that some factors or conditions can complicate your work with migrant youth ?

- Could you tell us about that ?
- Is this relevant to migrants in general ?

1. Do you have any suggestions to change or improve your encounters with migrants ?

- Probe : do you need any specific training, support… ?
- What about migrant youth ?

**Participants’ views of the experiences of migrants when seeking and using sexual and reproductive health services.**

1. What do you think about the way the health system is handling migrants’ needs ?

Probe : do you think that the health system is able to meet migrants’ needs ?

What about migrant youth’s needs ?

Do you think that they are receiving equal care?

1. According to previous studies migrant women in Sweden face several barriers (language for example) when accessing sexual and reproductive health services. Is that something you have experienced ?

- How ?
- What about migrant youth’s experiences ?

1. Could you tell us more about migrants’ needs when seeking SRH services ?

- Is there any specific group that needs more attention ?
- What about migrant youth ?

1. Do you want to add something?
2. Is there something that you thought that we would ask about it and we didn’t?

Thank you for your participation in this study.

**Appendix 2: Analysis process**

**Preliminary themes**

**Final themes and sub-themes**
